# Do ADHD-impulsivity and BMI have shared polygenic and neural correlates?

**DOI:** 10.1038/s41380-019-0444-y

**Published:** 2019-06-21

**Authors:** Edward D Barker, Alex Ing, Francesca Biondo, Tianye Jia, Jean-Baptiste Pingault, Ebba Du Rietz, Yuning Zhang, Barbara Ruggeri, Tobias Banaschewski, Sarah Hohmann, Arun L. W Bokde, Uli Bromberg, Christian Büchel, Erin Burke Quinlan, Edmund Sounga-Barke, April B. Bowling, Sylvane Desrivières, Herta Flor, Vincent Frouin, Hugh Garavan, Philip Asherson, Penny Gowland, Andreas Heinz, Bernd Ittermann, Jean-Luc Martinot, Marie-Laure Paillère Martinot, Frauke Nees, Dimitri Papadopoulos-Orfanos, Luise Poustka, Michael N Smolka, Nora C. Vetter, Henrik Walter, Robert Whelan, Gunter Schumann, IMAGEN Consortium

**Affiliations:** 1 Institute of Psychiatry, Psychology and Neuroscience, King’s College London, London, UK; 2 Centre for Population Neuroscience and Stratified Medicine (PONS), MRC Social, Genetic and Developmental Psychiatry (SGDP) Centre, London, UK; 3 Institute of Science and Technology for Brain-Inspired Intelligence, Fudan University, Shanghai, China; 4 Key Laboratory of Computational Neuroscience and Brain-Inspired Intelligence, Ministry of Education, Fudan University, Shanghai, China; 5 Division of Psychology & Language Sciences, University College London, London, UK; 6 Department of Child and Adolescent Psychiatry and Psychotherapy, Central Institute of Mental Health, Medical Faculty Mannheim, Heidelberg University, Square J5, 68159, Mannheim, Germany; 7 Discipline of Psychiatry, School of Medicine and Trinity College Institute of Neuroscience, Trinity College Dublin, Dublin, Ireland; 8 University Medical Centre Hamburg-Eppendorf, House W34, 3.OG, Martinistr. 52, 20246 Hamburg, Germany; 9 School of Health Science, Merrimack College, 315 Turnpike Street North Andover, North Andover, MA 01845 USA; 10 Department of Cognitive and Clinical Neuroscience, Central Institute of Mental Health, Medical Faculty Mannheim, Heidelberg University, Square J5, Mannheim, Germany; 11 Department of Psychology, School of Social Sciences, University of Mannheim, 68131 Mannheim, Germany; 12 Departments of Psychiatry and Psychology, University of Vermont, 05405 Burlington, VT USA; 13 Sir Peter Mansfield Imaging Centre School of Physics and Astronomy, University of Nottingham, University Park, Nottingham, UK; 14 Charité - Universitätsmedizin Berlin, corporate member of Freie Universität Berlin, Humboldt-Universität zu Berlin, and Berlin Institute of Health, Department of Psychiatry and Psychotherapy, Campus Charité Mitte, Charitéplatz 1, Berlin, Germany; 15 Physikalisch-Technische Bundesanstalt (PTB), Abbestr. 2 - 12, Berlin, Germany; 16 Institut National de la Santé et de la Recherche Médicale, INSERM Unit 1000 “Neuroimaging & Psychiatry”, University Paris Sud, University Paris Descartes - Sorbonne Paris Cité; and Maison de Solenn, Paris, France; 17 Institut National de la Santé et de la Recherche Médicale, INSERM Unit 1000 “Neuroimaging & Psychiatry”, University Paris Sud, University Paris Descartes; Sorbonne Université; and AP-HP, Department of Child and Adolescent Psychiatry, Pitié-Salpêtrière Hospital, Paris, France; 18 Department of Child and Adolescent Psychiatry and Psychotherapy, University Medical Centre Göttingen, von-Siebold-Str. 5, 37075 Göttingen, Germany; 19 Department of Psychiatry and Neuroimaging Center, Technische Universität Dresden, Dresden, Germany; 20 Department of Psychiatry and Psychotherapy, Technische Universität Dresden, Dresden, Germany; 21 School of Psychology and Global Brain Health Institute, Trinity College Dublin, Dublin, Ireland; 22 Present Address: NeuroSpin, CEA, Université Paris-Saclay, F-91191 Gif-sur-Yvette, France

**Keywords:** Psychology, Psychiatric disorders

## Abstract

There is an extensive body of literature linking ADHD to overweight and obesity. Research indicates that impulsivity features of ADHD account for a degree of this overlap. The neural and polygenic correlates of this association have not been thoroughly examined. In participants of the IMAGEN study, we found that impulsivity symptoms and body mass index (BMI) were associated (*r* = 0.10, *n* = 874, *p* = 0.014 FWE corrected), as were their respective polygenic risk scores (PRS) (*r* = 0.17, *n* = 874, *p* = 6.5 × 10^−6^ FWE corrected). We then examined whether the phenotypes of impulsivity and BMI, and the PRS scores of ADHD and BMI, shared common associations with whole-brain grey matter and the Monetary Incentive Delay fMRI task, which associates with reward-related impulsivity. A sparse partial least squared analysis (sPLS) revealed a shared neural substrate that associated with both the phenotypes and PRS scores. In a last step, we conducted a bias corrected bootstrapped mediation analysis with the neural substrate score from the sPLS as the mediator. The ADHD PRS associated with impulsivity symptoms (*b* *=* 0.006, 90% CIs = 0.001, 0.019) and BMI (*b* *=* 0.009, 90% CIs = 0.001, 0.025) via the neuroimaging substrate. The BMI PRS associated with BMI (*b* *=* 0.014, 95% CIs = 0.003, 0.033) and impulsivity symptoms (*b* *=* 0.009, 90% CIs = 0.001, 0.025) via the neuroimaging substrate. A common neural substrate may (in part) underpin shared genetic liability for ADHD and BMI and the manifestation of their (observable) phenotypic association.

## Introduction

Attention deficit hyperactivity disorder (ADHD) is a neurodevelopmental disorder with a typical onset during childhood, but also shows mixed patterns of continuity and remittance, along with an adulthood onset [1]. Previous studies indicate an association between adult ADHD and being overweight and, *in extremis*, obesity [2, 3]—conditions which are relatively common and have marked long-term health effects [4, 5]. Research has identified factors contributing to the comorbidity between ADHD and obesity, including  fetal programming,  psychosocial stress, and factors directly related to energy balance, reduced physical exercise and sleep patterns alterations [6]. The bulk of current research has focused on how neuropsychological impairments (e.g., impulsivity) associated with ADHD may increase dysregulated and excessive eating [2, 3]. Importantly, neural and genetic liabilities may be important components underlying the phenotypic association between ADHD and overweight/obesity [6].

With regard to neural correlates, a recent ADHD ENIGMA mega-analysis [7] reported smaller subcortical volumes for ADHD (*n* = 20,183) compared to controls (n = 35,191) in the accumbens, amygdala, caudate nucleus, hippocampus and putamen. Importantly, some of these brain areas are involved in inhibitory control and reward-related pathways [7]. Neuroimaging studies in individuals who are overweight or obese have identified similar regions implicated in inhibitory control and reward-related pathways [8], including the amygdala, hippocampus and prefrontal cortex as well sensory areas (precentral gyrus) that could relate to food cues [9]. Genome wide association studies (GWAS) have identified susceptibility loci for ADHD [10] and obesity [11]. A polygenic risk score (PRS) – a cumulative genetic risk profile from across the genome – derived from the ADHD GWAS [10] showed associations with obesity-related indices of body mass index (BMI [12]), waist-to-hip ratio, childhood obesity, HDL cholesterol and Type 2 diabetes. Another study [13] using the ADHD PRS reported positive associations with BMI, depression and substance use, with an inverse relationship with numeric reasoning, suggesting genetic overlap with neurocognitive performance (see also [14]). Indeed, with regard to neurology, loci from the ADHD GWAS [10] have been associated with both ADHD risk and lower intracranial volume, as well as the amygdala, caudate nucleus and putamen [15].

PRS approaches have been applied to brain imaging studies. Although this has yet to be done with ADHD and obesity simultaneously, a PRS based on the recent ADHD GWAS [10] associated with smaller caudate volume [16]. The use of PRS in schizophrenia has been associated with abnormalities in both brain structure [17] and function [18]. With regard to structure, Neilson et al. [17] reported an association with bilateral frontal gyrification, which is a hypothesized neural endophenotype of schizophrenia [19].

These findings suggest that a better understanding of the relationship between ADHD and overweight/obesity may be gained by using an approach that incorporates both genetic and neural variation. We used the rich dataset of the ongoing IMAGEN study, comprising impulsivity symptoms, BMI, genome-wide loci and whole brain structural and functional neuroimaging measures at age 19. Firstly, we aimed to examine associations between PRS scores and phenotypes for ADHD and BMI, to assess genetic and phenotypic relationships between these traits. Secondly, we aimed to assess the associations between the PRS scores for ADHD and BMI, whole-brain structural variation, as well as a functional neuroimaging measure that assesses reward-related impulsivity. We were also interested in examining the degree to which the PRS scores of ADHD and BMI associated with the phenotypic measurements of impulsivity symptoms and BMI via the identified neural imaging substrate (a mediational model). We had no a priori hypotheses about associations between the PRS scores and brain regions; we ran the analyses in an exploratory manner.

## Methods

### Participants

We conducted all analyses on individuals drawn from the IMAGEN study (www.imagen-europe.com), a large-scale imaging genetic study aimed at identifying genetic, neuroimaging, and behavioural bases of individual variability in psychiatric disorders. Genetic data was derived from blood samples collected at the age of 14, while clinical, physical and neuroimaging data analysed in the present study were collected at the age of 19. Psytools software (Delosis Ltd, London, UK) was used to conduct the behavioural characterization via its internet-based platform. The assessment battery of questionnaires and cognitive tasks was self-administered both in participants’ homes and at the neuroimaging facilities. The study was approved by local ethics research committees at each site. Written informed consent was obtained from all participants as well as from their legal guardians. Measures.

*Impulsivity symptoms* at age 19 were assessed using self-reported scores taken from the well-validated Barratt Impulsivity Scale (BIS) [20]. The BIS is a questionnaire that is widely used to assess trait-impulsivity in a variety of studies including ADHD [21], neuroimaging [22], personality [23], over-eating [24] and in relation to genetic influence and criminal behaviours [25]. At the time the study was conducted, the IMAGEN consortium had BIS data for 1319 individuals at 19 years-old.

### Body mass index (BMI)

BMI at age 19 was derived from height and weight measurements. Height was measured using a standard protocol to the last complete centimeter, weight was measured to the nearest 100 g. BMI was calculated as weight (kg)/height(m^2^). BMI values were recorded for 1347 subjects at the time the study was conducted. We excluded 114 participants who either were underweight (BMI < 18.5) extremely overweight (BMI > 50).

### Genetic data

After quality control, 1982 cases were included in our sample totalling 506,932 SNPs available for PRS. Population stratification was controlled for in all analyses via the use of the first eight principal components of the genetic data, which were used as covariates. Full details on acquisition and initial processing are given in the supplementary materials of this paper.

### MRI data

In this investigation, we used voxel-based morphometry measures of grey matter derived from T_1_ weighted MRI acquisitions and activation maps derived from the Monetary Incentive Delay (MID) task, to examine neural responses to reward anticipation and reward outcome. Full details on the MRI acquisitions [26] pre-processing [27] and confounds used in the analysis are given in the supplementary materials of this paper.

*A Task Based fMRI Acquisition of the Monetary Incentive Delay* (MID) task was used to examine neural responses to reward anticipation and reward outcome [28]. The task consisted of 66 10-s trials. In each trial, participants were presented with one of three cue shapes (cue, 250 ms) denoting whether a target (white square) would subsequently appear on the left or right side of the screen and whether 0, 2 or 10 points could be won in that trial. After a variable delay (4000–4500 ms) of fixation on a white crosshair, participants were instructed to respond with left/right button-press as soon as the target appeared. Feedback on whether and how many points were won during the trial was presented for 1450 ms after the response. Using a tracking algorithm, task difficulty (i.e., target duration varied between 100 and 300 ms) was individually adjusted such that each participant successfully responded on ~66% of trials. Based on prior research suggesting reliable associations between ADHD symptoms and fMRI blood-oxygen-level dependent (BOLD) responses measured during reward anticipation, the current study used the contrast ‘anticipation of high-win vs anticipation of no-win’.

### Statistical analyses

The analyses proceeded in three steps (code used in statistical analyses is available upon request). In Step 1, PRS scores were created for ADHD and BMI and we examined associations between the scores and phenotypic measures. We also calculated associations between the ADHD and BMI PRS scores themselves, in order to investigate a potential genetic comorbidity between ADHD and BMI.

To derive the ADHD PRS, summary statistics were downloaded from the Psychiatric Genomics Consortium (http://www.med.unc.edu/pgc/results-and-downloads; 20,183 cases and 35,191 controls, all European descent). To calculate the BMI PRS, GWAS summary statistics from 339,224 European descents were downloaded from the GIANT Consortium (http://portals.broadinstitute.org/collaboration/giant/index.php). Deriving a PRS necessitates the use of a significance threshold for inclusion of SNPs in the calculation of the score (e.g., all SNPs associated at *p* < 0.05). In investigations where the researcher is only interested in the relation between the score and the phenotype, it is possible to vary that threshold, and select that which results in the highest variance explained between the PRS and the phenotype [29, 30]. However, in the present investigation, we were also interested in both the PRS scores themselves, and in their relation to functional and structural neuroimaging measures. In this case, varying the threshold for SNP inclusion in the PRS scores for ADHD and BMI, respectively, can lead to overfitting on the phenotype of interest [30]. Given the nature of our research question, which involves multiple phenotypes and multiple brain markers, this overfitting could lead to circularity problems in further analyses [31]. Using multiple PRS scores also poses a problem with multiple comparisons: if several PRS scores are calculated on the same phenotype, a correction must be carried out over these tests to ensure significance [30]. We therefore chose to use a nominal threshold of *p* = 0.05 for the inclusion of SNPs in the calculation of the score. This approach avoids both overfitting and multiple testing. All genetic data processing and analyses were performed using the R package PRSice [15] and PLINK [17]. In order to minimise the statistical assumptions necessary for valid inference, all significance levels reported here were ascertained using permutation testing. As we are explicitly looking for comorbidities between different data types, and the sPLS analysis only identifies positive associations, all significance tests were one-sided.

In Step 2, we investigated whether ADHD and BMI share a common underlying neural substrate. We used a sparse formulation of partial least squares (sPLS) for this purpose [18, 19]. This method is designed to establish associations between multiple sets of variables by finding the weighted sum of variables in each set, which correlate maximally with the weighted sum of variables that are connected via a path diagram (see Supplementary Figure S1). We used five-fold cross-validation to quantify the strength and significance of the associations of individual PRS scores and phenotypes, with the VBM and MID measures they were connected to via the path diagram [32].

Although PLS methods are very powerful, results can be difficult to interpret, as all variables contribute to associations between data-views. This is particularly problematic with neuroimaging data, which is high dimensional. It would be useful to know which neuroimaging features were associated with PRS scores and phenotypes of impulsivity and BMI. For this reason, we used a method that induces sparsity by setting some PLS weights to zero through the application of an L_1_ penalty, applied under resampling [33, 34]. Through this approach, one can identify weighted sets of brain regions that are associated with the PRS scores and phenotypes. This set of brain regions may therefore be considered as an endophenotype for ADHD and BMI. We used a resampling procedure to ensure that the PLS approach only retained features that were robustly associated with phenotypes and genetic measures [33]. Significance was ascribed using a permutation testing procedure. A detailed description of the exact analysis approach used is detailed in the Supplementary Materials. In all analyses, we controlled for genetic population stratification (i.e., the 8 principle components), gender, imaging site, age and total intracranial volume. Many association tests were carried out in this investigation. We corrected for multiple comparisons using the Holm-Bonferroni method [35].

In Step 3, we examined the degree to which the set of brain regions identified by sPLS may act as an intermediary endophenotype between the PRS scores and the impulsivity and BMI phenotypes. In other words, we examined if the brain regions could help explain (or mediate) the observable association between ADHD and BMI genetic vulnerabilities and the impulsivity and BMI phenotypes. The mediation (or indirect) pathways were defined by the product term of the pathways of interest (e.g., PRS *to* brain BY brain *to* phenotype). There were four overall possible effects: 1) ADHD PRS → brain regions → impulsivity symptoms; 2) ADHD PRS → brain regions → BMI; 3) BMI PRS →brain regions → BMI; and 4) BMI PRS →brain regions → impulsivity. Because standard errors underlying mediation pathways (i.e., the product terms) are known to be skewed, we bootstrapped all indirect effects 10,000 times with bias corrected (90% and 95%) confidence intervals. The mediation pathways reported here are based on the bootstrapped variability around the product of non-standardized path coefficient estimates (i.e., *b*). Mediation pathways were programmed in Laavan [36] in the statistical package R [37].

## Results

### Step 1: PRS scores for ADHD and BMI

At the time the study was conducted, *n* = 874 subjects had complete genetic and phenotypic data. The BMI PRS score was significantly associated with BMI (*r* = 0.23, *n* = 874, *p* = 2.2 × 10^−11^ FWE corrected) and the PRS score for ADHD was significantly associated with impulsivity symptoms (*r* = 0.10, *n* = 874, *p* *=* 0.014 FWE corrected). We also note that impulsivity symptoms were associated with BMI (*r* = 0.10, *n* = 874, *p* = 0.014 FWE corrected), and that the PRS scores for ADHD and BMI were significantly cross-correlated (*r* = 0.17, *n* = 874, *p* = 6.5 × 10^−6^, FWE corrected). Of interest, using Steiger’s test for dependent correlations [38], we found that the correlation between the PRS scores of ADHD and BMI was higher (*p* = 0.036 FWE corrected) than the correlation between the phenotypic measures of impulsivity and BMI.

### Step 2: the shared neural correlates of the PRS scores of ADHD and BMI

**Fig. 1:**
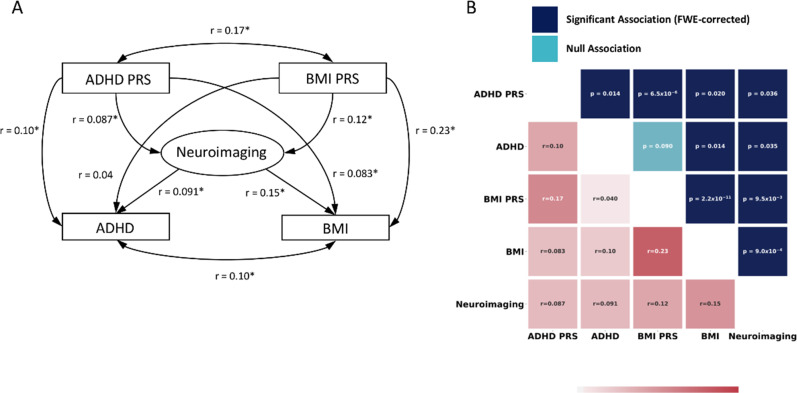
**a** The panel on the left shows the path diagram illustrating associations between the different biological and phenotypic measures used in this investigation. Associations that are statistically significant are marked with an asterisk. **b** The panel on the right shows correlation values between the different biological and phenotypic measures investigated in this study, the lower triangular matrix shows correlation values between the various biological measures, whilst the upper triangular matrix shows the FWE-corrected significance levels of these associations

Of the 874 subjects who had both complete genetic and phenotypic data, 604 had both T_1_ and fMRI data that passed QC. Using sPLS, we found that both the ADHD and BMI phenotypes, and their respective polygenic risk scores, are significantly associated with a common neural substrate, constructed from T_1_ and fMRI data, which we term the ‘neural endophenotype’. The PRS for BMI was associated with the neural endophenotype at r = 0.12, *n* = 604, *p* = 9.5 × 10^−3^, FWE corrected, whilst the ADHD PRS was significant at *r* = 0.087, *n* = 604, *p* = 0.036 FWE corrected. ADHD and BMI phenotypes were also associated with this set of brain regions, with ADHD associated at *r* = 0.091, *n* = 604, *p* = 0.035 FWE corrected, and BMI associated at *r* = 0.15, *n* = 604, *p* = 9.0 × 10^−4^ FWE corrected respectively. These correlations are summarized in a matrix and path diagram in Fig. 1.

**Fig. 2:**
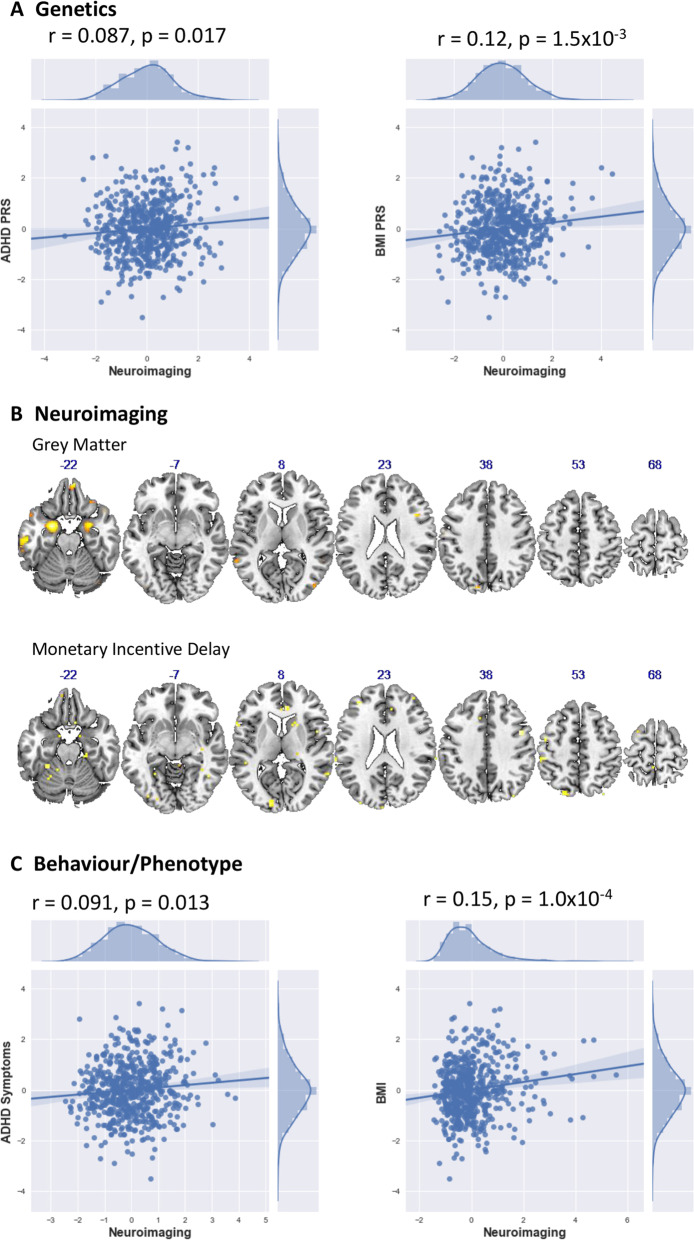
**a** The scatter plots in panel (**a**) of the figure show relations between neuroimaging and ADHD and BMI PRS scores. **b** The brain regions in panel (**b**) of the figure illustrate the VBM and MID regions that are associated with ADHD and BMI PRS scores, and their associated phenotypes. **c** The scatter plots in panel (**c**) of the figure show relations between neuroimaging and ADHD and BMI phenotypes

The neural endophenotype is made up of grey matter regions and regions of activation derived from the MID task. Grey matter regions contributing to this neural endophenotype were located bilaterally in the cerebellum, amygdala, hippocampus, para-hippocampus and orbital frontal cortex, which all present bilaterally. There was also a largely left lateralised grey matter cluster in the inferotemporal cortex. sPLS weights in each of these brain regions were negative, implying an inverse association between PRS scores/phenotypes and grey matter. Neural endophenotype clusters that came up in the MID task were largely left lateralised and included the fusiform gyrus and para-hippocampus, postcentral and parietal inferior, calcarine and occipital superior and frontal superior medial cortex. These results are displayed in Fig. 2. sPLS weights in each of these brain regions were positive, implying a positive association between the MID contrast map and the PRS/phenotype scores. The full set of VBM and MID clusters identified and localised in the sPLS analysis are tabulated in Supplementary Tables S1 and S2.

### Step 3: Neural endophenotype as a mediator in the link between PRS scores and ADHD and BMI

In Step 3, we saved the neuroimaging factor score from the sPLS and conducted the multivariate bias corrected bootstrapped mediation analyses. The four mediation pathways were observed, albeit at a 90% CIs for ADHD and 95% CIs BMI, suggesting that the neural endophenotype may act as an intermediary variable in the link between the PRS scores and the impulsivity and BMI phenotypes. These four mediation pathways were: 1) ADHD PRS → neuroimaging score → impulsivity (*b* *=* 0.006, 90% CIs = 0.001, 0.019); ADHD PRS → neuroimaging score → BMI (*b* *=* 0.009, 90% CIs = 0.001, 0.023); 3) BMI PRS → neuroimaging score → BMI (*b* *=* 0.014, 95% CIs = 0.003, 0.033); and 4) BMI PRS → neuroimaging score → impulsivity (*b* *=* 0.009, 95% CIs = 0.001, 0.025).

## Discussion

This study set out to examine if there are shared genetic and neural correlates of the ADHD (impulsivity) and BMI [39] phenotypes. Our results support previous ADHD-related research in showing an association between these two phenotypes [3]. Further analyses showed that the PRS scores for ADHD and BMI were correlated with each other and with grey matter across the brain and contrast maps derived from fMRI scans conducted during the MID task. Importantly, these neuroimaging measures also associated with the phenotypes of impulsivity and BMI. These results extend present knowledge of the biological underpinnings of the association between ADHD and BMI in three main ways.

Firstly, using previously established genome-wide evidence, and a multi-modal whole-brain approach, this study showed that the PRS scores and phenotypes of ADHD and BMI simultaneously associated with decreases in grey matter in similar brain areas as reported by the case-control ADHD ENIGMA mega-analysis [7] as well as certain case-control designs of obesity [9]. These areas include the cerebellum, amygdala, hippocampus, orbitofrontal and inferotemporal cortex. The functions of these brain areas underscore the importance of impairments in cognitive and behavioural control and impulsivity in both ADHD and BMI. For example, the fronto-cerebellar networks have been implicated in impairments of cognitive control (e.g., over-riding impulses) in both ADHD and obesity [40, 41]; the ability to consider future consequences/adjust behaviours [41]; and the inhibition of eating behaviour [42]. Likewise, the amygdala and hippocampus have been associated with impulsivity symptoms in ADHD [43] as well as weight gain and over-eating behaviours such as continuing to eat even when sated [44–47]. Finally, the orbital frontal cortex has been linked to impairments in cognitive and attentional control in ADHD [48], but also potentially to selection and consumption of calorie-rich foods [49] and sensitized reactivity to (food-related) reward [50]. The second set of findings relate to the reward anticipation fMRI task. There was an increased BOLD response for the posterior visual and association areas, postcentral, cerebellum and frontal medial cortex. This was consistent with previous findings from both the MID task and other fMRI paradigms used to assess reward processes. With regard to the MID task, our findings converged with previous research that reported abnormalities in frontal-parietal and cerebellar-parietal networks [51, 52]. Supporting other reward processing studies, we identified increased BOLD response in the cerebellum and postcentral gyri [47, 53], in addition to reward areas in the medial frontal regions [54, 55]. Importantly, both the MID-related results and previously mentioned grey matter results showed abnormalities in visual processing areas (i.e., inferotemporal and association cortices for grey matter and posterior visual and association cortices for the MID task). These abnormalities may be implicated in visual attention biases to reward-related cues (e.g., food or reward) in both obesity and ADHD [37–39].

Third, we examined a multivariate mediation model. Here, the associations between the ADHD and BMI PRS scores and the impulsivity and BMI phenotypes were partially carried by the brain areas identified by sPLS. We therefore suggest that these brain areas (structural + functional) may act as a neural endophenotype [56] between the genetic liabilities of ADHD and obesity and the manifestation of their (observable) phenotypic association. By definition neurobiological endophenotypes mark genetic vulnerability but are also independent of disease state [57]. Our suggestion of a neural endophenotype is consistent with the idea that a limited number of neural systems may engender risk for a range comorbid psychiatric syndromes [56]. As such, this hypothesized neural endophenotype might extend as vulnerability for known comorbidities of ADHD and BMI, such as substance use and addiction [58] and depression [59, 60], to name a few.

How might the results of the present study bear on future research? Are there (potential) clinical implications? It is important to note that our findings of genetic influence on ADHD and BMI are non-deterministic and can be context dependent [61]. Indeed, Barcellos et al. [57], taking advantage of a natural experiment, showed that an additional year of compulsory schooling in the UK benefitted individuals with higher genetic risk for obesity, reducing the gap in unhealthy body size in the top and bottom terciles of genetic risk of obesity from 20 to 6 percentage points. This is an interesting result when it is considered that certain interventions targeting ADHD can result in weight reduction and, potentially, vice-versa. Although findings are mixed, studies on Methylphenidate show that a reduction in ADHD symptoms can associate with decreased BMI, especially for overweight/obese adolescents [3, 62]. Methylphenidate has also been proposed as a treatment for obesity [63, 64]. Future research should examine if individuals at high genetic risk for both ADHD and BMI will especially benefit from interventions tailored for one—or both—of these conditions.

Our findings should be considered in light of a number of limitations. Firstly, our models were statistically driven—although theoretically informed. The models are therefore dependent on the properties of the measures included. While our sPLS model identified simultaneous association between the PRS scores, the two neuroimaging modalities and the phenotypic measurements of ADHD and BMI, this does not mean that this model—or the study variables contained within it—is the best (or only) solution. Secondly, it is also important to note that PRS and neural correlates only explain a small proportion of the liability for many psychiatric difficulties and other traits. Thirdly, the current findings differ from reward anticipation in case-control ADHD designs [65, 66] and for clinical remission [67]. Relatedly, it will be of interest to replicate the present results in clinical samples, for ADHD and BMI separately, across different age groups, and across different ethnic groups. Fourth, in the mediation analyses, the ADHD-related pathway had larger bias corrected CIs (90%) compared to the BMI-related pathway (95%). There are at least two reasons for these different CIs. The GWAS discovery sample for ADHD (*n* = 46,350) is smaller than BMI (*n* = 339,224), which can affect the relative predictive power of these PRS scores [68]. In addition, we examined the ADHD PRS in relation to impulsivity symptoms, which is less precise than examining the BMI PRS in relation to BMI.

## Conclusion

Our results indicate that ADHD and BMI PRS scores are significantly correlated, suggesting shared genetic risk between the traits. In a data-driven model, both the PRS scores were associated with the phenotypic measures of ADHD and BMI, as well as shared neural correlates of impairments in inhibitory control and reward processing.

## Supplementary information

Supplemental Material
