# Supplementary material for: Do ADHD-impulsivity and BMI have shared polygenic and neural correlates?
Source: Mol Psychiatry. 2019 Jun 21;26(3):1019–28. doi: 10.1038/s41380-019-0444-y (PMC7910212; doi:10.1038/s41380-019-0444-y)
Supplement: Supplementary file 1 — Supplemental Material [file 41380_2019_444_MOESM1_ESM.docx]

**Supplementary Materials**

**Do ADHD-Impulsivity and BMI have shared polygenic and neural correlates?**

**Materials and Methods………………………………………………………………………………………………2**

Genetic Acquisition…………………………………………………………………………………………………….2

MRI Acquisition..…………………………………………………………………………………………………………3

Partial Least Squares Analysis..………………………………………………………………………………….5

**Supplementary Figures**

Figure S1……………………………………………………………………………………………………………………11

**Supplementary Tables…………………………………………………………………………………………….13**

Table S1…………………………………………………………………………………………………………………….12

Table S2…………………………………………………………………………………………………………………….13

**References………………………………………………………………………………………………………………18**

**Materials and Methods**

**Genetic Acquisition**

DNA purification and genotyping was performed by the Centre National de Génotypage in Paris. DNA was purified from whole blood samples (~10 ml) preserved in BD Vacutainer EDTA tubes (Becton, Dickinson and Company) using Gentra Puregene Blood Kit (QIAGEN) according to the manufacturer’s instructions. Genotype information was collected at 582,982 markers using the Illumina HumanHap610 Genotyping BeadChip. Single nucleotide polymorphisms with call rates of < 98%, minor allele frequency < 2% or deviation from the Hardy-Weinberg equilibrium (*p* ≤ 1×10^-4^) were excluded from the analyses. Individuals with an ambiguous sex code, excessive missing genotypes (failure rate > 2%), and outlying heterozygosity (heterozygosity rate 3 standard deviations from the mean) were also excluded. Identity-by-state similarity was used to estimate cryptic relatedness for individual using PLINK software^1^ . Closely related individuals with identity-by-descent (IBD > 0.1875) were eliminated from the subsequent analysis. Population stratification for the genome-wide association study data was examined by principal component analysis (PCA) using EIGENSTRAT software. The four HapMap populations were used as reference groups in the PCA and individuals with divergent ancestry (from CEU) were also excluded. Ten MDS components were also downloaded from IMAGEN, and were used as covariates in the analyses. Genetic data was collected for 2091 subjects at baseline. Of these, 257 subjects failed QC, leaving 1,834 cases available for our sample, totaling 463,940 SNPs available for polygenetic-risk score (PRS) analysis.

**MRI Acquisition**

We sought to find a common neural substrate linking the polygenic risk of ADHD and BMI, and their actual phenotypic expression. We used T_1_ data, and fMRI data collected during a monetary incentive delay (MID) task for this purpose. T_1_ data is widely available and has previously used in studies of both BMI and ADHD symptoms^2^ ^3^ . We also used the MID task, which is designed to quantify reward anticipation^4^ - an important underlying component of of both overweight and ADHD symptoms. At the time of the analysis, 1423 subjects had MID and VBM data.

**T_1_ Weighted MRI Acquisition**

Scanning took place at eight different sites across Europe, using scanners built by four different manufacturers (Siemens, Philips, General Electric, Bruker). High resolution, T_1_ weighted images were obtained using a Magnetization Prepared Rapid Acquisition Gradient Echo (MPRAGE) sequence, based on the ADNI protocol (http://www.loni.ucla.edu/ADNI/Cores/index.shtml). Scan parameters were standardized across sites to the highest degree possible (sagittal slice plane; repetition time: 2.3 s; echo time 2.8 ms; flip angle 8°; 256×256×160 matrix; isotropic voxel size: 1.1 mm).

**VBM Pre-processing**

Scans were preprocessed using both the Computational Anatomy Toolbox (CAT12) running in SPM12^5^ . Given the young adults recruited in IMAGEN, we first used CAT12 in order to avoid using adult tissue probability maps (TPM) to initiate the segmentation process^6^. The CAT12 toolbox segmentation relies on an adaptive Maximum a Posterior technique and TPMs used in CAT12 are for registration purposes only. Diffeomorphic registration (Dartel) was then used to register the images, and to generate the study-specific population average template^6^ . We then resliced the data to 1.5x1.5x1.5mm voxel size. Smoothing was carried out using an isotropic 8 mm full width at half maximum Gaussian smoothing kernel. We created a mask for the sample by taking the mean across all VBM maps included in the sample and thresholding the image at > 0.4. We used a stringent mask to avoid overfitting the data^5^. Following a visual QC, 114 scans were not used as they were considered to be of insufficient quality.

**Task Based fMRI Acquisition**

FMRI data were acquired across 40 slices in descending order (2.4 mm, 1 mm gap) using a gradient-echo T2*-weighted EPI sequence (TR: 2200 ms;TE: 30 ms; 64x64 matrix; anterior-posterior commissure line plane). For the MID task, SST and FT, 300, 444 and 160 volumes were acquired for each subject, respectively.

**The Monetary Incentive Delay Task for fMRI**

Participants performed a modified version of the Monetary Incentive Delay (MID) task to examine neural responses to reward anticipation and reward outcome^4^ . The task consisted of 66 10-second trials. In each trial, participants were presented with one of three cue shapes (cue, 250 ms) denoting whether a target (white square) would subsequently appear on the left or right side of the screen and whether 0, 2 or 10 points could be won in that trial. After a variable delay (4,000-4,500 ms) of fixation on a white crosshair, participants were instructed to respond with left/right button-press as soon as the target appeared. Feedback on whether and how many points were won during the trial was presented for 1,450 ms after the response. Using a tracking algorithm, task difficulty (i.e. target duration varied between 100 and 300 ms) was individually adjusted such that each participant successfully responded on ~66% of trials. Participants had first completed a practice session outside the scanner (~5 minutes), during which they were instructed that for each 5 points won they would receive a reward.

Based on prior research suggesting reliable associations between ADHD-symptoms and fMRI BOLD responses measured during reward anticipation, the current study used the contrast ‘anticipation of high-win vs anticipation of no-win’. Only successfully ‘hit’ trials were included here. Following a QC of the behavioral task data, and the contrast maps derived from MRI data, 74 scans were not used as they were considered to be of insufficient data quality.

**Sparse Partial Least Squares
Partial Least Squares**

Partial Least Squares (PLS) path modelling is a method that can be used to establish relations between multiple ‘blocks’ of data^7^ ; used in this context, the term ‘data block’ denotes one or more variables that are used together as predictors when seeking to find relations to other data blocks. In this investigation, ADHD PRS, BMI PRS, ADHD symptoms, BMI, and VBM and MID data represent the different blocks we wish to relate. The first step of a PLS path modelling analysis is to define a set of ‘paths’ we wish to establish between different data blocks. These paths represent the predictions we wish to make between blocks. In the present context, we are interested in finding relations between polygenic risk scores, neuroimaging measures of brain structure and function, and BMI and ADHD symptoms. PLS is then designed to find the weighted sum of variables in each block of data, which correlate optimally with the weighted sum of variables in other blocks. Using the PLS method outlined here, we found relations between four different phenotypes, and two different neuroimaging modalities. We would like to understand how much overall variance is explained between polygenic risk scores, neuroimaging measures, and phenotypes. A naive way to do this would be to simply combine MID and VBM modalities into a single data block and treat them this way in a PLS analysis. However, treating neuroimaging data in this way is likely to lead to the VBM data overwhelming the MID data when finding associations between data blocks. This is because VBM data contains many more features than MID data. Instead, we follow a two-step approach, which is common in PLS path analyses. In the first step, we treat the VBM and MID data separately; this analysis is called the ‘inner loop’. The inner loop results in two neuroimaging vectors, one for MID, and one for VBM. These vectors are then combined in an ‘outer loop’^7^ . In the outer loop, we conduct a second PLS, using the outputs of the first PLS as inputs. Two-step procedures of this kind are often used in this kind of analysis. The analysis design can be illustrated using path diagrams. The path diagrams for the present analysis is displayed in Supplementary Figure S1.

**Sparsity and Stability Selection**

In the form originally proposed by Wold et al^7^ , latent variables are taken as the weighted sum across all variables in the pertinent block. This can make the interpretation of results difficult as all variables contribute to any significant multivariate relations we are able to establish between data views; In order to increase the interpretability of our results, we used a sparse formulation of the PLS framework. This formulation involves the application of an L_1_ penalty to PLS weight vectors, the L_1_ penalty forces weights that make a negligible contribution to the variance explained between sets, to exactly zero. This means that only a subset of the most important variables contribute to the relation between data views. This approach is termed sparse PLS (sPLS)^7^ ^8^ .

Although the L_1_ penalty discussed above is widely used to enforce sparsity in a multitude of methodological contexts, it has been shown to be unstable as a feature selection procedure in applications involving a large number of variables, particularly when these variables are multicollinear (as they are here). Stability selection is a general procedure that has been developed to address both of these issues^10^ . The essential idea behind stability selection is very simple: if data is repeatedly resampled, with some sparsity inducing procedure applied to each resampling, variables exhibiting a ‘real’ effect will be selected more often than noise. Therefore, stability selection methods require that we repeatedly resample the data, applying our selection algorithm to each resample. We then select variables for the final model that appear in a certain proportion of resamples, denoted here by S. In the present investigation, we applied stability selection using a hundred resamplings of the data (as suggested by Meinhausen et al^10^ ). We considered variables as being stable if they were present in over S = 0.75 of resamples (amounting to 0.75 x 100 = 75 resamples in the present scenario). We chose this fraction on the basis of prior literature on the subject: Meinhausen et al suggested using a cut-off fraction in the range of S = 0.6-0.9; we used a fraction of S = 0.75 as it is in the centre of this suggested range. Therefore, parameter selection pertaining to the stability selection procedure is quite clear. However, the approach specified here still requires that we choose a sparsity level to be applied via the L_1_ penalisation. It has been shown that simply setting the sparsity level at random over different resamplings of the stability selection procedure performs well. This approach has the advantage that it privileges the selection of variables that are not only stable across subjects, but also across different L_1_ penalty thresholds^10^ .

**Confounds**

It is important to consider the effects of confounding variables in PLS analyses as confounding sources of variance that are shared between data views can lead to spurious effects. In PLS analyses, confounds are normally dealt with by regressing covariates of no interest on different data views, and applying the PLS algorithm to the residuals^8^ . We used gender, site, age and total intracranial volume as covariates of no interest.

**Analysis Design**

The sPLS approach used here is non-parametric in nature. This means that it is not possible to ascertain the significance of any results obtained by comparing them to some known distribution. Instead, resampling and permutation testing methods must be used to determine statistical significance. We used five-fold cross validation to establish the strength of associations between the PRS scores, the endophenotype, and the ADHD and BMI phenotypes^11^ . We used permutation testing to determine the significance of these associations. The full analysis procedure is described sequentially in the text below. This information is also illustrated in Supplementary Figure S1.

1. BMI, ADHD and their PRS scores were z-normalised to have a mean of zero and a standard deviation of one. 
2. We extracted different neuroimaging measures. These measures were transformed to n x p matrices. Here, n is the sample size and p is the number of features in the neuroimaging modality under consideration.

3. We generated cross-validation indices. In this process, the data was randomly split into five segments, to be used iteratively for training and testing.
4. The first of the cross-validation segments was then taken as the test set. The remaining four fifths of the data then constituted the training set.

5. Stability selection procedure: The training data was then resampled a 100 times. The sPLS algorithm was applied to each resampling of the data. Sparsity values for each of the neuroimaging measures, in each resampling, was set at random between 1 and $\surd p$, where p is the number of features in the relevant data view.

6. We then took the variables (in the two neuroimaging data views) that were retained in over 75 of the resamples (stability selection), and re-applied the sPLS algorithm to these variables, this time without inducing sparsity in the process given that this has been already established via the stability selection procedure. This step resulted in two vectors, one for each neuroimaging measure, which were each optimized to correlate with each one of the following: ADHD phenotype, BMI phenotype. ADHD PRS score, BMI PRS score. This is the inner PLS loop optimization and is displayed in figure S1 beside the workflow diagram.

7. We then applied a second sPLS analysis, linking these neuroimaging measures with the BMI and ADHD phenotypes vectors, and their associated PRSs. This is the outer PLS loop optimisation and is displayed in figure S1 beside the workflow. In this step of the PLS analysis, we did not enforce sparsity in any of the data views.

8. We then applied the sPLS weights estimated in the previous step to the test set.

9. Steps 4-8 were then repeated five times, once for each of the cross-validation folds.
10. We then permuted the data and repeated steps 3-9 10,000 times, in order to determine the significance of associations between the two PRSs, the endophenotype, and the phenotypes themselves.

The procedure outlined in the text above results in five different sPLS models, one for each cross-validation fold. This could complicate interpretation. For this reason, following this analysis procedure, we then applied steps 5-7 to the whole data-set to generate a single model. It is this model that is discussed in the manuscript.

The present investigation involved a large number of comparisons. We controlled the family-wise error rate over these multiple comparisons using the Bonferroni-Holm Procedure^12^ .


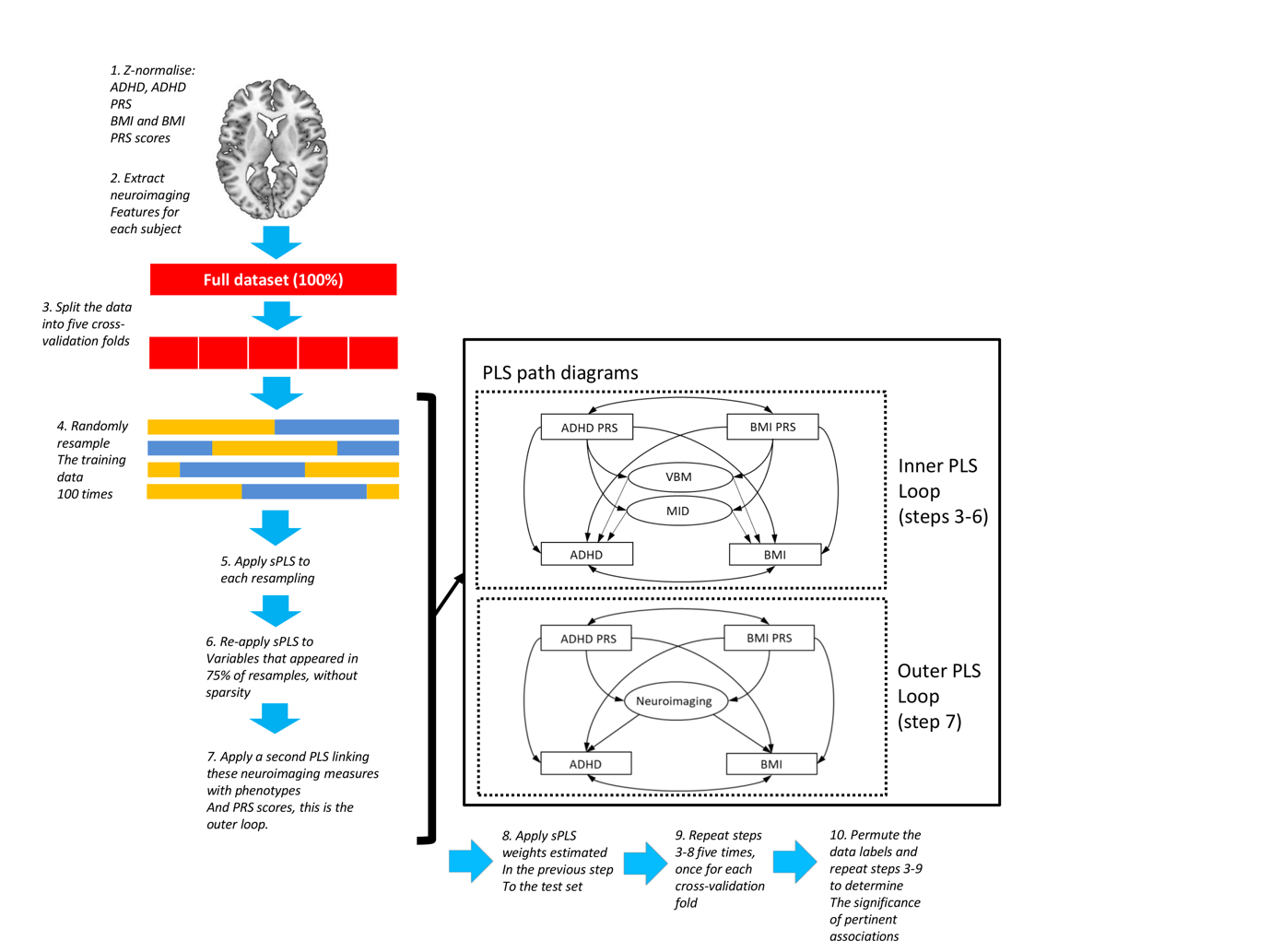


*Figure S1: This figure illustrates the analysis pipeline for the sPLS analysis. The main workflow is shown on the left whilst path diagrams illustrating the relations between different data types are shown on the right.*

| **x** | **y** | **z** | **Cluster**  **Size** | **Regions Covered** |
| --- | --- | --- | --- | --- |
| 22 | -67 | -56 | 780 | Cerebelum_7b_R, Cerebelum_8_R |
| -22 | -1 | -21 | 707 | Olfactory_L, Hippocampus_L, ParaHippocampal_L, Amygdala_L, Temporal_Pole_Sup_L |
| -60 | -20 | -24 | 400 | Temporal_Mid_L, Temporal_Inf_L |
| -33 | -75 | -13 | 355 | Occipital_Mid_L, Occipital_Inf_L, Fusiform_L, Cerebelum_6_L |
| 3 | 58 | -1 | 319 | Frontal_Sup_Medial_L, Frontal_Sup_Medial_R, Frontal_Med_Orb_L, Frontal_Med_Orb_R, Cingulum_Ant_L |
| 27 | 0 | -20 | 314 | Hippocampus_R, ParaHippocampal_R, Amygdala_R, Temporal_Pole_Sup_R |
| 46 | 14 | 28 | 226 | Precentral_R, Frontal_Inf_Oper_R, Frontal_Inf_Tri_R |
| 31 | 38 | -17 | 164 | Frontal_Mid_Orb_R, Frontal_Inf_Orb_R |
| -21 | -72 | -55 | 131 | Cerebelum_7b_L, Cerebelum_8_L |
| -47 | -10 | 11 | 130 | Rolandic_Oper_L, Insula_L, Postcentral_L, Heschl_L |
| 51 | 11 | -4 | 96 | Rolandic_Oper_R, Insula_R, Temporal_Pole_Sup_R |
| -14 | -85 | 34 | 91 | Cuneus_L, Occipital_Sup_L |
| -52 | 13 | -20 | 85 | Temporal_Pole_Sup_L, Temporal_Mid_L, Temporal_Pole_Mid_L |
| 30 | -62 | 47 | 64 | Occipital_Sup_R, Angular_R |
| 6 | 53 | -22 | 60 | Frontal_Sup_Orb_R, Rectus_R |
| -60 | -47 | 3 | 59 | Temporal_Mid_L |
| -62 | -24 | 19 | 59 | Postcentral_L, SupraMarginal_L, Temporal_Sup_L |
| 21 | 60 | -12 | 53 | Frontal_Sup_Orb_R, Frontal_Mid_Orb_R |
| 13 | -22 | 11 | 46 | Thalamus_R |
| -58 | -12 | 34 | 45 | Postcentral_L |
| -7 | -70 | 27 | 44 | Calcarine_L, Cuneus_L |
| 55 | -16 | -32 | 40 | Temporal_Inf_R |
| -7 | -79 | 3 | 27 | Calcarine_L, Lingual_L |
| 44 | -81 | 10 | 16 | Occipital_Mid_R |
| 37 | -87 | -3 | 11 | Occipital_Inf_R |
| -15 | 21 | -24 | 6 | Frontal_Sup_Orb_L |
| 9 | 4 | -11 | 5 |  |
| -50 | -70 | -14 | 4 | Occipital_Inf_L |
| 14 | 17 | -12 | 4 | Rectus_R |
| -28 | -92 | 17 | 3 | Occipital_Mid_L |
| 23 | 13 | -35 | 2 | Temporal_Pole_Mid_R |
| 41 | -77 | -21 | 2 | Cerebelum_Crus1_R |
| -48 | -73 | -16 | 2 | Occipital_Inf_L |
| 6 | 2 | -10 | 2 |  |
| -7 | -93 | 25 | 2 | Cuneus_L |
| 21 | 9 | -32 | 1 | ParaHippocampal_R |
| 50 | 20 | -21 | 1 | Temporal_Pole_Sup_R |
| -51 | -44 | -18 | 1 | Temporal_Inf_L |
| -18 | -75 | -11 | 1 | Lingual_L |
| -18 | -75 | -8 | 1 | Lingual_L |
| -9 | 51 | 0 | 1 | Cingulum_Ant_L |
| -8 | -95 | 21 | 1 | Cuneus_L |

*Table S1: Grey matter clusters, derived from T_1_ data, pre-processed using VBM, which were found to be significantly associated with BMI, ADHD, and BMI and ADHD PRS scores. Co-ordinates refer to locations in MNI space.*

| **x** | **y** | **z** | **Cluster Size** | **Regions Covered** |
| --- | --- | --- | --- | --- |
| -27 | -42 | -16 | 75 | ParaHippocampal_L, Lingual_L, Fusiform_L, Precuneus_L, Temporal_Inf_L, Cerebelum_4_5_L, Cerebelum_6_L |
| -49 | -34 | 49 | 52 | Postcentral_L, Parietal_Inf_L, SupraMarginal_L |
| -12 | -93 | 0 | 40 | Calcarine_L, Occipital_Sup_L, Occipital_Mid_L, Occipital_Inf_L |
| -5 | 30 | 32 | 35 | Frontal_Sup_Medial_L, Cingulum_Ant_L, Cingulum_Mid_L |
| 45 | 2 | 40 | 28 | Precentral_R, Frontal_Inf_Oper_R |
| 29 | -87 | 32 | 27 | Occipital_Sup_R, Occipital_Mid_R |
| -20 | -73 | 48 | 26 | Parietal_Sup_L, Parietal_Inf_L |
| 5 | 58 | -18 | 19 | Frontal_Sup_Orb_R, Rectus_L, Rectus_R |
| 17 | 13 | 4 | 18 | Caudate_R, Putamen_R, Pallidum_R |
| -37 | -13 | -30 | 16 | Fusiform_L, Temporal_Inf_L |
| 47 | -55 | -12 | 15 | Fusiform_R, Temporal_Inf_R |
| -27 | -84 | -12 | 14 | Lingual_L, Occipital_Inf_L, Fusiform_L |
| -26 | -97 | 12 | 14 | Occipital_Mid_L |
| -9 | 57 | -19 | 12 | Frontal_Sup_Orb_L, Rectus_L |
| 48 | 6 | 10 | 12 | Rolandic_Oper_R |
| -36 | 42 | 21 | 12 | Frontal_Mid_L |
| 19 | -75 | 41 | 12 | Cuneus_R, Occipital_Sup_R, Precuneus_R |
| -8 | 5 | 60 | 11 | Supp_Motor_Area_L |
| 7 | 37 | 8 | 10 | Cingulum_Ant_L, Cingulum_Ant_R |
| 8 | 36 | 25 | 10 | Cingulum_Ant_R |
| -26 | -56 | -26 | 9 | Cerebelum_6_L |
| -22 | -34 | -21 | 9 | ParaHippocampal_L, Fusiform_L, Cerebelum_4_5_L |
| -12 | -81 | -6 | 9 | Lingual_L |
| -55 | -52 | 0 | 9 | Temporal_Mid_L |
| 23 | 55 | 17 | 9 | Frontal_Sup_R |
| -1 | -53 | -28 | 8 | Vermis_4_5, Vermis_9, Vermis_10 |
| -32 | -91 | 22 | 8 | Occipital_Mid_L |
| -32 | -2 | -32 | 7 | ParaHippocampal_L, Fusiform_L |
| 24 | -26 | -22 | 7 | ParaHippocampal_R, Fusiform_R, Cerebelum_4_5_R |
| 19 | -30 | -7 | 7 | Hippocampus_R, ParaHippocampal_R, Lingual_R |
| 11 | 54 | 28 | 7 | Frontal_Sup_Medial_R |
| -42 | 3 | 34 | 7 | Precentral_L |
| -46 | -4 | 53 | 7 | Precentral_L |
| 4 | -66 | 65 | 7 | Parietal_Sup_R, Precuneus_R |
| -54 | -5 | -39 | 6 | Temporal_Inf_L |
| 12 | 17 | -21 | 6 | Frontal_Sup_Orb_R, Rectus_R |
| -55 | -42 | 5 | 6 | Temporal_Mid_L |
| 62 | -53 | 7 | 6 | Temporal_Mid_R |
| 61 | -31 | 47 | 6 | SupraMarginal_R |
| -24 | 5 | 64 | 6 | Frontal_Sup_L, Frontal_Mid_L |
| -38 | -49 | -32 | 5 | Cerebelum_Crus1_L, Cerebelum_6_L |
| -43 | -56 | -16 | 5 | Fusiform_L |
| -7 | -71 | 5 | 5 | Lingual_L |
| -65 | -32 | 21 | 5 | SupraMarginal_L, Temporal_Sup_L |
| 38 | 51 | 24 | 5 | Frontal_Mid_R |
| -53 | -20 | 29 | 5 | Postcentral_L |
| 53 | 18 | 29 | 5 | Frontal_Inf_Oper_R |
| -43 | -77 | -10 | 4 | Occipital_Inf_L |
| -59 | 14 | 9 | 4 | Frontal_Inf_Oper_L |
| 47 | -80 | 14 | 4 | Occipital_Mid_R |
| 6 | -19 | 12 | 4 | Thalamus_R |
| 18 | -85 | 45 | 4 | Cuneus_R |
| 11 | -36 | 47 | 4 | Cingulum_Mid_R |
| 29 | -78 | 52 | 4 | Parietal_Sup_R |
| -2 | 2 | 69 | 4 | Supp_Motor_Area_L |
| 35 | -8 | -41 | 3 | Fusiform_R |
| -41 | 1 | -30 | 3 | Temporal_Mid_L, Temporal_Inf_L |
| -14 | -46 | -21 | 3 | Cerebelum_4_5_L |
| 32 | -96 | 12 | 3 | Occipital_Mid_R |
| -32 | 37 | 11 | 3 | Frontal_Inf_Tri_L |
| -7 | 37 | 11 | 3 | Cingulum_Ant_L |
| -15 | -96 | 24 | 3 | Occipital_Sup_L |
| -3 | 12 | 25 | 3 | Cingulum_Ant_L |
| -53 | 16 | 28 | 3 | Frontal_Inf_Oper_L, Frontal_Inf_Tri_L |
| -55 | -2 | 34 | 3 | Precentral_L |
| 33 | 3 | 60 | 3 | Frontal_Mid_R |
| 49 | -20 | 63 | 3 | Postcentral_R |
| -1 | -45 | 68 | 3 | Precuneus_L, Precuneus_R |
| 56 | 1 | -38 | 2 | Temporal_Inf_R |
| -29 | -57 | -35 | 2 | Cerebelum_6_L |
| 5 | -55 | -35 | 2 | Vermis_9 |
| -3 | -66 | -31 | 2 | Vermis_8 |
| 36 | -48 | -32 | 2 | Cerebelum_Crus1_R |
| 44 | 8 | -29 | 2 | Temporal_Mid_R, Temporal_Pole_Mid_R |
| -23 | -79 | -23 | 2 | Cerebelum_Crus1_L |
| 12 | -78 | -20 | 2 | Cerebelum_Crus1_R, Cerebelum_6_R |
| 38 | 34 | -20 | 2 | Frontal_Inf_Orb_R |
| 18 | 52 | -20 | 2 | Frontal_Mid_Orb_R |
| 24 | -36 | -17 | 2 | Fusiform_R |
| -18 | 49 | -17 | 2 | Frontal_Sup_Orb_L |
| 36 | -36 | -14 | 2 | ParaHippocampal_R, Fusiform_R |
| -36 | -84 | -7 | 2 | Occipital_Inf_L |
| -29 | -52 | -8 | 2 | Fusiform_L |
| 23 | -58 | -5 | 2 | Lingual_R |
| 5 | -49 | -4 | 2 | Vermis_4_5 |
| 6 | -39 | -5 | 2 | Vermis_3 |
| -11 | 20 | -5 | 2 | Caudate_L |
| 8 | 71 | -4 | 2 | Frontal_Sup_Orb_R, Frontal_Med_Orb_R |
| 6 | -79 | -1 | 2 | Lingual_R |
| -45 | -72 | -2 | 2 | Occipital_Inf_L |
| -18 | 62 | 6 | 2 | Frontal_Sup_L |
| -48 | -30 | 10 | 2 | Temporal_Sup_L |
| 36 | -24 | 10 | 2 | Heschl_R |
| 35 | -91 | 18 | 2 | Occipital_Mid_R |
| -39 | -13 | 18 | 2 | Rolandic_Oper_L, Insula_L |
| 38 | -88 | 21 | 2 | Occipital_Mid_R |
| -5 | -97 | 24 | 2 | Cuneus_L |
| 6 | -40 | 24 | 2 | Cingulum_Post_R |
| -36 | 31 | 25 | 2 | Frontal_Inf_Tri_L |
| -57 | -6 | 45 | 2 | Postcentral_L |
| 23 | 8 | 48 | 2 | Frontal_Sup_R, Frontal_Mid_R |
| 9 | -54 | 49 | 2 | Precuneus_R |
| -35 | -58 | 64 | 2 | Parietal_Sup_L |
| 26 | -61 | 67 | 2 | Parietal_Sup_R |
| -33 | -10 | -41 | 1 | Fusiform_L |
| 39 | -46 | -38 | 1 | Cerebelum_Crus1_R |
| -39 | -7 | -38 | 1 | Fusiform_L |
| 42 | -43 | -35 | 1 | Cerebelum_Crus1_R |
| -36 | 5 | -35 | 1 | Temporal_Inf_L |
| 54 | 20 | -35 | 1 | Temporal_Pole_Mid_R |
| -48 | -64 | -32 | 1 | Cerebelum_Crus1_L |
| 27 | 5 | -29 | 1 | ParaHippocampal_R |
| 36 | 8 | -26 | 1 | Temporal_Pole_Sup_R |
| 15 | -1 | -23 | 1 | ParaHippocampal_R |
| -12 | -1 | -23 | 1 | ParaHippocampal_L |
| 66 | -46 | -20 | 1 | Temporal_Inf_R |
| 3 | 14 | -20 | 1 | Olfactory_R |
| -39 | 29 | -20 | 1 | Frontal_Inf_Orb_L |
| 18 | 32 | -20 | 1 | Frontal_Sup_Orb_R |
| 3 | -52 | -17 | 1 | Vermis_4_5 |
| -6 | 14 | -17 | 1 | Olfactory_L |
| -42 | 32 | -17 | 1 | Frontal_Inf_Orb_L |
| -15 | 62 | -17 | 1 | Frontal_Mid_Orb_L |
| 45 | -46 | -11 | 1 | Temporal_Inf_R |
| 24 | -52 | -8 | 1 | Lingual_R |
| 36 | -46 | -8 | 1 | Fusiform_R |
| -9 | -37 | -8 | 1 | Cerebelum_4_5_L |
| -24 | -25 | -8 | 1 | Hippocampus_L |
| 36 | -19 | -8 | 1 | Hippocampus_R |
| -51 | -19 | -8 | 1 | Temporal_Mid_L |
| 0 | 8 | -8 | 1 | Olfactory_L |
| 30 | -7 | -5 | 1 | Putamen_R |
| 30 | 11 | -5 | 1 | Putamen_R |
| -54 | -37 | -2 | 1 | Temporal_Mid_L |
| -45 | -52 | 1 | 1 | Temporal_Mid_L |
| -9 | -13 | 1 | 1 | Thalamus_L |
| 12 | -67 | 4 | 1 | Lingual_R |
| -24 | -52 | 4 | 1 | Precuneus_L |
| -6 | -82 | 7 | 1 | Calcarine_L |
| -48 | -37 | 7 | 1 | Temporal_Mid_L |
| 21 | -25 | 10 | 1 | Thalamus_R |
| 48 | -7 | 10 | 1 | Heschl_R |
| -48 | -79 | 13 | 1 | Occipital_Mid_L |
| 66 | -37 | 16 | 1 | Temporal_Sup_R |
| -12 | -1 | 16 | 1 | Caudate_L |
| -24 | 53 | 16 | 1 | Frontal_Mid_L |
| -39 | 2 | 19 | 1 | Insula_L |
| -42 | 50 | 19 | 1 | Frontal_Mid_L |
| 69 | -31 | 22 | 1 | Temporal_Sup_R |
| 30 | -91 | 25 | 1 | Occipital_Sup_R |
| -18 | 11 | 25 | 1 | Caudate_L |
| -36 | 14 | 25 | 1 | Frontal_Inf_Tri_L |
| 27 | -70 | 37 | 1 | Occipital_Sup_R |
| 66 | -28 | 40 | 1 | SupraMarginal_R |
| 54 | 5 | 40 | 1 | Precentral_R |
| -30 | -49 | 43 | 1 | Parietal_Inf_L |
| -3 | -10 | 43 | 1 | Cingulum_Mid_L |
| -33 | 8 | 43 | 1 | Precentral_L |
| 48 | 17 | 43 | 1 | Frontal_Mid_R |
| 36 | 26 | 43 | 1 | Frontal_Mid_R |
| 33 | 38 | 43 | 1 | Frontal_Mid_R |
| 9 | 8 | 46 | 1 | Cingulum_Mid_R |
| 36 | -76 | 52 | 1 | Parietal_Sup_R |
| 36 | -13 | 52 | 1 | Precentral_R |
| -9 | -19 | 58 | 1 | Paracentral_Lobule_L |
| 54 | -13 | 58 | 1 | Precentral_R |
| 30 | -13 | 58 | 1 | Precentral_R |
| -18 | 5 | 58 | 1 | Frontal_Sup_L |
| 24 | 11 | 64 | 1 | Frontal_Sup_R |
| -6 | -67 | 67 | 1 | Precuneus_L |
| 36 | -58 | 67 | 1 | Parietal_Sup_R |
| 36 | -4 | 70 | 1 | Frontal_Sup_R |

*Table S2:* *Activation clusters, derived from the large-win vs no-win contrast of the MID task carried out under fMRI scanning, which were found to be significantly associated with BMI, ADHD, and BMI and ADHD PRS scores in the sPLS analysis. Co-ordinates refer to locations in MNI space.*

**References**

1. Purcell S, Neale B, Todd-Brown K, et al. PLINK: A tool set for whole-genome association and population-based linkage analyses. *The American journal of human genetics*. 2007;81(3):559-575.

2. Hoogman M, Bralten J, Hibar DP, et al. Subcortical brain volume differences in participants with attention deficit hyperactivity disorder in children and adults: A cross-sectional mega-analysis. *The Lancet Psychiatry*. 2017;4(4):310-319.

3. Medic N, Ziauddeen H, Ersche KD, et al. Increased body mass index is associated with specific regional alterations in brain structure. *Int J Obes*. 2016;40(7):1177.

4. Knutson B, Westdorp A, Kaiser E, Hommer D. FMRI visualization of brain activity during a monetary incentive delay task. *Neuroimage*. 2000;12(1):20-27.

5. Penny WD, Friston KJ, Ashburner JT, Kiebel SJ, Nichols TE. *Statistical parametric mapping: The analysis of functional brain images.* Elsevier; 2011.

6. Ashburner J, Friston KJ. Unified segmentation. *Neuroimage*. 2005;26(3):839-851.

7. Wold H. Partial least squares. *Encyclopedia of statistical sciences*. 2004;9.

8. Monteiro JM, Rao A, Shawe-Taylor J, Mourão-Miranda J, Alzheimer's Disease Initiative. A multiple hold-out framework for sparse partial least squares. *J Neurosci Methods*. 2016;271:182-194.

9. Witten DM, Tibshirani R, Hastie T. A penalized matrix decomposition, with applications to sparse principal components and canonical correlation analysis. *Biostatistics*. 2009;10(3):515-534.

10. Meinshausen N, Bühlmann P. Stability selection. *Journal of the Royal Statistical Society: Series B (Statistical Methodology)*. 2010;72(4):417-473.

11. Friedman J, Hastie T, Tibshirani R. *The elements of statistical learning.* Vol 1. Springer series in statistics New York; 2001.

12. Holm S. A simple sequentially rejective multiple test procedure. *Scandinavian journal of statistics*. 1979:65-70.
